# MARGRET M. AND PAUL B. BALTES FOUNDATION IN BEHAVIORAL AND SOCIAL GERONTOLOGY AWARD LECTURE

**DOI:** 10.1093/geroni/igz038.844

**Published:** 2019-11-08

**Authors:** Nancy Pedersen

**Affiliations:** Karolinska Institutet, Stockholm, Sweden

## Abstract

The Margret M. and Paul B. Baltes Foundation Award in Behavioral and Social Gerontology recognizes outstanding early-career contributions in behavioral and social gerontology. The lecture will be given by the 2018 Baltes Award recipient, Frank Infurna, PhD, of Arizona State University. This session will also include the presentation of the 2019 Baltes Award. The 2019 Baltes Award recipient is Allison Bielak, PhD, of Colorado State University. Supported by the Margret M. and Paul B. Baltes Foundation.

